# Epidemic Cholera at New Orleans

**Published:** 1849-03

**Authors:** 


*Epidemic Cholera at New Orleans.* By E. D. FENNER, M. D.—The following history of the late epidemic visitation of cholera at New Orleans, was written for publication in the New Orleans Med. and Surg. Journal, of which Dr. Fenner was formerly one of the Editors. Not being prepared, however, in season for that Journal, Dr. F. consented to its insertion in the New Orleans Picayune, a copy of which he has kindly forwarded to us. We have perused the letter with much interest, feeling assured from our personal knowledge of the author, that a report could not emanate from one more capable and responsible. We commend the article to our readers, to all of whom, we doubt not, it will be, at the present time, most acceptable. EDITOR BUFF. MED. JOURNAL.

*To the Editor of the New Orleans Medical and Surgical Journal.*

DEAR SIR:—According to your request, I shall offer you some brief memoranda of the epidemic cholera which has recently scourged our city. We have passed through a long-dreaded and most dangerous crisis; and now that "the action" is over, and the dust and smoke of the "battle field" (if you will allow the metaphor) are cleared away, it is both meet and proper for us to review the scene, take account of the "killed and wounded," and endeavor to learn from the result some lesson of wisdom and usefulness. Nor do I deem my *military* metaphor altogether inappropriate to the present time. True, our city is shrouded in mourning and sadness, yet many have escaped the peril of death; and, as we have recently commemorated our almost miraculous deliverance from the arms of the invader in days of yore, let us not be unmindful or ungrateful for our recent deliverance from an impending danger scarcely less terrific. We have encountered an *unseen and a dreadful foe*—one whose progress was marked by the victims strewn along his course. Yet even his ravages were not unmixed with mercy. Here and there a victim was overwhelmed, as by an avalanch, and there was no help for him; but for the most part, fair and timely warning was given, and those who attended to the dictates of wisdom and prudence, found but little difficulty in escaping the impending danger. Amidst the general alarm and distress that pervaded the community, the duties and responsibilities which devolved upon the respectable portion of the medical profession were of the most serious and important nature: they were met and performed with a firmness and fidelity worthy of a passing notice. Without regarding the unjust and illiberal imputations that were cast upon the profession, it is not to be denied that the physicians of New Orleans have boldly stood their ground, shared the common danger, and done all in their power, as well to instruct their fellow-citizens how to keep well, as to rescue them when ill. What better evidence need I adduce to substantiate this assertion, than the fact that although nearly everybody felt more or less the epidemic influence, there were comparatively but few bad cases and very few deaths amongst the better classes of people—such as usually apply to respectable and educated physicians for medical aid? The reason is obvious: these people applied for medical aid in good season—they obtained the best advice and remedies, and were promptly cured; whilst others were either deluded into false security, by relying upon some worthless but well-puffed nostrum, or through ignorance and temerity neglected all remedies until the disease had advanced beyond the curable stage. This was the penalty of ignorance and folly—and a severe one, too. Without farther preliminaries, let us note some of the more prominent facts connected with the rise, progress and results of the epidemic.

The commencement of the late epidemic may be dated from the 11th of December, when the ship "Swanton" arrived at this port, thirty-nine days from Havre, with 280 steerage passengers, consisting of German and French immigrants—chiefly German. Now, whether it be a mere coincidence that epidemic cholera broke out in this city just at the time when a vessel arrived having some cases of cholera on board, or that said vessel brought the infection, which rapidly spread through the whole community, is an exceedingly debatable question. But let me go on with a statement of such facts and circumstances as I have, before I attempt to debate it. The whole subject is replete with interest. Everything connected with it is new to me, and I will endeavor to make the most rational induction in my power, having no preconceived theory to substantiate.

For several weeks previous to the arrival of the "Swanton," the weather had been changeable—for the most part very warm, though there had been several white frosts. Yellow fever had almost disappeared, and there was but little sickness prevailing; though amongst the existing diseases were observed some remarkable cases of stomach and bowel complaints. On the 5th of December I attended a gentleman on Custom-house street, who labored under vomiting, pain and spasms in the bowels, and prostration to such a degree that, if epidemic cholera had been supposed to be here, no person would have hesitated to pronounce him a case. He had no rice water evacuations, but his bowels were rather costive, and he vomited bile; but many such cases have been seen since the epidemic was declared. He recovered after two or three days' illness, and has not been again sick.

Some days previous to this, three or four negroes were attacked with cholera morbus on the same night and at the same house, in Gravier street. They were promptly treated, and all soon recovered. Similar cases were observed in the practice of a number of physicians in different parts of the city, all going to show, as it appears to me, that the epidemic influence of cholera was gradually being matured and developed in our midst.

I have recently learned some other facts, which are worthy of notice in connection with the commencement of this epidemic. The ship "Güttemburg," from Hamburg, with some 250 steerage passengers, after a passage of fifty-five days, arrived at New Orleans on the 6th of December. Cholera was prevailing at Hamburg when this ship left, and six or seven deaths from it occurred on board before she got out of the Elbe. As soon as the vessel got out to sea, the disease subsided completely, and no more cases occurred during the whole voyage. As there were no cases of cholera on board when she arrived here, it attracted no attention, although she came from an infected port; but I am informed by one of the visiting physicians of the Charity Hospital, that soon after the epidemic broke out here, a man died in one of his wards, who stated that he had recently arrived from Germany, on board a vessel which had lost several passengers by cholera. What became of the other passengers of the "Guttemburg" I know not.

In addition to this I should not omit the following fact, obtained from the records of the Mayor's office and the newspapers of the day, viz: The bark "Callao," from Bremen, having 152 German emigrants on board, after a passage of forty-eight days, arrived at New Orleans on the 8th of December, was anchored off Slaughter House Point, on the opposite side of the river. The Secretary of the Board of Health was sent to examine her on the 11th of December, and reported that: "During the voyage eighteen of the immigrants died, some of whom died with *purging and vomiting*, and others with violent attacks of diarrhœa. The last death occurred on the 30th of November. At present no cases of sickness on board, and those who left the vessel since its arrival are well. N. B.—It is reported in the log-book that the first case that died perished from cholera. This is merely the opinion of those on board, and is not entitled to much weight."

The Callao remained over on the opposite side of the river until about the fourth of January, when she was brought over on this side to be loaded.

The ship Swanton left Havre on the 2d or 3d of November. *There was no cholera at Havre when she left*, nor have we heard of any there since. There was none in any part of France; but the epidemic had reached Germany, and some of the passengers on board the Swanton were German emigrants. Whether they came from an infected district or not, we are not informed. The vessel was out twenty-six days before a death occurred, the first being from consumption on the 28th November. We learn that sixteen or seventeen deaths occurred during the passage, most of them from bowel complaints, supposed to be dysentery. The Swanton reached New Orleans on the 11th of December, and took position at the wharf in the *upper part of the Second Municipality*. On the morning of the 12th a woman was carried from the ship to the Charity Hospital, and found to be in a complete state of collapse. She was reported to have been attacked the night previous with violent vomiting, purging and cramps.

The intelligent house surgeon, Dr. Wedderstrandt, as well as a number of other physicians who saw this case, at once recognised it as Asiatic cholera, and the Board of Health was notified of the fact. The woman died at 6 P. M. The Secretary of the Board, Dr. Hester, was immediately despatched for the purpose of examining the condition of the vessel and passengers. He reported the facts above stated, and in addition, that "he found two old women laboring under bowel complaints, and two children suffering from debility—the ship in a good condition as regards cleanliness—passengers generally look well."

On the morning of the 13th, a man who came over on the same vessel, was brought to the Charity Hospital, and found to be in a complete state of collapse. He was cold and pulseless, but his intellect was perfectly clear, and he gave the following account of himself: He said he had a slight diarrhœa on the morning of the 11th, but he walked about the city and ate an apple. On the 12th he left the ship and went to a boarding-house near the Poydras Market—still had slight diarrhœa, but ate no fruit this day. After going to bed, was attacked with severe vomiting, purging and cr mps—took no medicine, and was reduced to a state of collapse when he entered the hospital. He died about 6 P. M. The books of the hospital show three other cases of cholera admitted on this day, all of which terminated fatally. They were from different parts of the city, and not passengers of the Swanton. On the same day I observed two women in the hospital from the same ship. They had only slight diarrhœa and were promptly relieved. The two fatal cases were seen by a number of physicians, most of whom felt no hesitation in pronouncing them Asiatic Cholera, though a different opinion was expressed by some. The rumor soon spread throughout the city and created great consternation.

On the evening of the same day (13th December) that the second case was taken to the Charity Hospital, a man who has resided here for many years, and who does business not far from the St. Charles Hotel, came into my office with strong symptoms of cholera. He had not been near the ship Swanton, nor seen any of the passengers. I prescribed for him, and on visiting him at his room, half an hour afterwards, I found him extremely ill, with severe pain in his bowels, copious watery purging, skin bathed in cold sweat, great thirst and general prostration. His condition was so alarming, and he derived so little relief from large and repeated doses of opium, calomel, camphor and capsicum, assisted by sinapisms, stimulating frictions, &c., that I determined to resort to the inhalation of chloroform. By this means he was made perfectly easy in about two minutes, and remained so until the other medicines he had taken had time to act. He got through the night pretty well, and recovered in a few days from a dangerous illness.

On the 14th December the Board of Health held a special meeting, and issued a card which appeared in the newspapers the following morning, assuring the public that there was no foundation for the rumor that the Asiatic cholera had made its appearance in the city. This statement was seconded by flourishing editorials in several of the newspapers of the day.

On the 15th December there were eight cases of cholera admitted into the Charity Hospital, and I heard of cases in the private practice of a number of physicians.

In a letter from a learned physician of this city to a distinguished professor in Paris, which was published in the Commercial Times of the 23d inst., the author mentions three fatal cases of cholera that occurred on Custom-house, Bienville and Chartres streets on the 15th. He goes on to say: "It is well enough to remark here, that these three primary victims of cholera in New Orleans were all cooks, going every morning, very early, to the principal market in the city, situated on the bank of the river, a cable's length from the infected vessel." In the latter part of this statement, the worthy author must have made a mistake, for the President of the Board of Health was informed by its Secretary, who was sent to examine the Swanton, that he found her in the upper end of the Second Municipality, which is nearly a mile from the aforesaid principal market, frequented by the unfortunate cooks. So these cases must have originated in a different way.

On the 16th December there were eleven cases of cholera admitted into the hospital, and the disease was evidently rapidly increasing in private practice.

On this day I was called to see Dr. J. B. Morgan, of Jackson, Miss., who was attacked the night previous, without having committed any other indiscretion than eating some fish and oysters at dinner. When I arrived at his room, I met Dr. Farrell, who had seen him before, and had good reason to be provoked at the difficulty he found in convincing Dr. Morgan of the danger he was in, and the importance of prompt and vigorous treatment. Dr. M. had already passed about thirty liquid evacuations, then had cramps in his legs, and in fact was on the verge of collapse. Dr. M. being an old friend and neighbor of mine, I joined my entreaties to the arguments of Dr. F., and we did all we could to convince him of the importance of vigorous treatment, but all to no purpose. He insisted that he was not dangerously ill—that he had been similarly affected many a time before, and that if he were not disturbed he would soon be well. The sequel verified our worst apprehensions. He was incorrigibly obstinate, dallied too long with a dangerous disease, and was lost.

The panic now prevailed throughout the city, and vast numbers of people fled in every direction; yet some of the leading newspapers, and a few physicians, hooted at the idea that the disease was *Asiatic Cholera*, and the Board of Health still kept aloof. From this time the disease increased so rapidly that on the 22d December, ten days from the time when the first case was admitted into the hospital, the number of deaths by cholera in that institution amounted to twenty-two, and in the whole city to forty-five. But let me not encroach upon your province, Mr. Editor. Being Secretary to the Board of Health, you will of course supply your journal with all the mortuary statistics that may be interesting to the profession. I may be permitted to state in this connection, however, that the Board of Health published on the morning of the 23d December that Asiatic cholera was "epidemic" in the city, the number of deaths from it the day previous having been forty-five; and they announced the cessation of the epidemic on the 6th of January, when the deaths amounted to thirty-eight.

The epidemic raged most severely from the 22d to the 30th of December, having reached its zenith about the 28th, on which day the deaths by cholera were ninety-two. From the 16th to the 22d the weather was oppressively warm, the thermometer rising as high as 84°. From the 22d it was cool, wet, and gloomy, till the night of the 30th, when there fell a white frost. On the morning of the 1st January there was another white frost, and from that time the disease declined steadily.

The epidemic influence appeared to be felt by almost every person in the city, whether native or foreigner, acclimated or unacclimated. Thousands complained of an extraordinary uneasiness in the stomach and bowels, but in a vast majority of instances it was easily relieved, and but few bad cases occurred amongst those who were prudent, and paid proper attention to the premonitory symptoms. The lower classes of people have evidently suffered most, which may be attributed to ignorance or neglect. The mortality at the Charity Hospital has been very great; yet no one can be surprised at it who visited the institution during the epidemic, and witnessed the condition in which the patients were when admitted. Cholera is an insidious disease that generally steals upon its victims, seldom declaring itself openly until it has them completely within its fatal grasp. I have not a doubt that seven-tenths of the people who have recently perished of it in this city might have been saved, if they had procured proper medical aid at the onset of the disease. I presume there is hardly a physician in the city who has not been called to persons reduced to the most dangerous condition by relying too implicitly and too long upon some of the various "specifics" advertised in our newspapers, and lauded by the editors. Yet some may have been saved by these very nostrums; for there are many in this goodly Commonwealth who have such an antipathy to physic, that they will not take it under any circumstances, unless they see its assumed virtues blazoned before the public by the effulgent illumination of fictitious puffs and certificates. They have a decided *penchant* for the marvellous, the mysterious, and the unknown. They scorn reason and common sense, and have contempt for honest simplicity in scientific researches. Like the followers of the veiled prophet of Khorassan, they

"Would be dupes and victims—and they are."

But let me not weary your patience with matters of this sort. The people are free agents, and have the right to take what medicine they like. If they prefer artful humbuggery to honest, unpretending science, why let them have it to their heart's content.

The mortality from Cholera at its late visitation, compares most favorably with that of 1832, when it first scourged our city. The number of deaths by cholera from the 12th December, 1848, to the 20th Jan., 1849, as appears from the reports of the Board of Health, amounts to near 1400, 596 of which occurred at the Charity hospital. We learn from an interesting Memoir on the Cholera of 1832, addressed to the Academy of Medicine of Paris, by Dr. M. Halphen, a French practitioner of this city at that time, that the disease made its appearance about the 25th of October, in the midst of an epidemic of yellow fever; that in a few days it raged severely, and that in the short space of twenty days it killed about 6000 people. Dr. Halphen says, that the mortality amounted on some days as high as 500 a day. He estimates the full population of the city then at 50,000, and as cholera broke out during the prevalence of yellow fever, ere yet the absent citizens had returned, and before the customary visitors dared to come in, he does not think the population at that time exceeded 35,000; thus showing the frightful loss of about *one-sixth of the people in about twenty days.* When we read over these sad details, we may well congratulate ourselves upon our happy deliverance from the late pestilence. True, we have lost about 1400 people, amongst them a few valuable citizens; but what would have been our fate if so malignant a disease as that of 1832 had broken out in December last, when all our own people were at home, and the city was full of strangers? In 1832 *the living could not afford decent burial to the dead.* Dr. Halphen states, that on some days upwards of one hundred corpses were accumulated at the cemeteries, waiting for interment. Large trenches were dug, into which cart-loads of uncoffined bodies were heaped indiscriminately; and in the dead of night, a great number of bodies, with bricks and stones tied to the feet, were stealthily thrown into the river. The same ratio of mortality at the present time would demand about twenty thousand victims. Let us turn from the apalling calculation, and thank God that we have been so mercifully spared.

As in 1832, the epidemic has declined to a stage of comparative security, but the disease has not entirely disappeared. There is as little cholera in New Orleans at the present time, in proportion to the population, as in any other part of the Lower Mississippi Valley. Whether the epidemic will be rekindled, at the approach of the ensuing summer, remains to be seen. If the miserable condition of the city, as regards cleanliness, will have any influence upon the event, we may certainly expect it. New Orleans must ever continue to be a prey to the most fatal diseases that prevail, until something efficient is done to improve its sanatory condition.

The manner in which the Cholera has spread from this city, in every direction, forms a problem as curious and difficult as that of its first appearance. Almost every vessel that left the city, a few days after the disease commenced, soon had cases aboard, and on some of the steamboats going up the river there were twenty or thirty cases and many deaths. Thus, persons having the disease, and dying of it, were carried to all the landing towns and cities up the river as high as Cincinnati. In many of these places it spread to a limited extent among the inhabitants; in others it did not. We have as yet heard of no place up the river where the disease has prevailed as an *epidemic*. We learn that cholera is spreading among the plantations along the river, and also in the interior of Louisiana. To some of these the infection appeared to be directly carried; at others it began without any communication with an infected district. The most remarkable mortality that we have heard of, out of the city of New Orleans, occurred in the 8th Infantry, a body of 450 soldiers which arrived here from Jefferson Barracks on the 1st of December, and were stationed at the Barracks, about four miles below New Orleans. There they remained till the 12th, when they embarked for Port Lavaca, in Texas, on board the steamships Telegraph and New Orleans. These ships reached Port Lavaca on the 15th, but the men did not land till the 20th December. On the night of the 21st, according to a correspondent of one of our newspapers, the right wing of the regiment, under the command of Brevet Major Gates, moved twelve miles into the country, the left wing, under command of Major Morrison, remaining in Lavaca. During the night the weather changed from sultry heat to a cold, rainy norther, and by daylight four soldiers of those left in the town were dead with cholera, and many laboring under the disease. On the following day an express came back from Major Gates, with the intelligence that his men were falling rapidly with the same disease. The disease raged with such severity that in the brief space of three or four days 115 men, or about one fourth of the command perished. Yet, strange as it may appear, the correspondent informs us that "no cases occurred among the citizens." Now these soldiers must have imbibed the morbific cause somewhere, which lay dormant in their systems, like a powerful enemy in ambush, until a fit opportunity was offered for action by the sudden and malign influence of a Texan norther. Then it sprung upon its unsuspecting victims, made dreadful havoc, and in a few days vanished.

We are informed that cholera has prevailed to a considerable extent at Houston, Texas, whilst Galveston, on the sea board, has escaped, although situated on the line of travel from New Orleans to Houston.

Soon after the epidemic commenced in this city, a trader on Esplanade street took his negroes (about sixty in number) across the lake and located them in the pine woods, where he hoped they would be perfectly secure. They were all well when they left the city, excepting one case, which terminated fatally on the day of their arrival over there, and continued well for nearly three weeks after reaching their point of destination. The cholera then broke out among them and killed a considerable number in a very short time.

At the Charity Hospital, probably as many as fifty cases have occurred among the nurses, servants and persons who had been admitted for other complaints.

After reviewing the few recent facts which I have just stated, what shall we say about the contagiousness or transportability of cholera? Numberless striking facts recorded in the history of cholera would seem to prove beyond cavil that it may be transported from place to place, through the medium of persons affected. On the other hand, the numerous instances in which the disease failed to be propagated through this medium, and the utter futility of rigid quarantine regulations and sanatory cordons in arresting its march, would seem to authorize a different opinion. Amidst these contending difficulties, if the reader can arrive at a satisfactory conclusion,

I can only say he is more fortunate than myself. Speaking of quarantine, perhaps we may hear before long that the city of Natchez, on the river above us, has been protected from cholera by *her* quarantine. I have been informed that there were some fatal cases of cholera in that place. Moreover, I have good authority for saying that the quarantine regulations of Natchez are altogether worthless, except to the officers charged with their enforcement.

I ought not to close this communication without saying something about the general character of the disease, and the treatment pursued by the physicians of New Orleans. As to the character of the epidemic, I think I may safely say that it has not been very malignant. In most instances the attack was insidious and mild—generally commencing with a looseness of the bowels, attended with more or less griping, and often accompanied by nausea and vomiting. The latter symptoms almost invariably attended those patients who had committed imprudence in eating. Without descending into minutiæ, I may say that the disease almost invariably commenced with some unusual disturbance of the digestive organs. When this disturbance commanded the attention it deserved, it was generally most easily remedied by the simplest means; but if neglected, it seldom failed to lead on to the most disastrous consequences. This, then, is the *curable stage* of cholera, and almost the only stage in which it can be cured; for if it be permitted to run on till the patient becomes cold and pulseless, ninety-nine in a hundred will inevitably die. By powerful means, reaction may often be established; but the danger is not yet passed—a great majority still die of the consecutive fever. Say what you will about creating *panic* and spreading alarm amongst the people, I feel no hesitation in asserting that when epidemic cholera is prevailing, every person who has any unusual diarrhœa, had better believe he is *a case* and act accordingly. If this simple rule was universally adopted, cholera would soon be rendered comparatively harmless. Thus, according to Dr. Watson, one of the ablest English authors, the disease was arrested in London by the establishment of "*Diarrhœa Dispensaries*," where the poor were supplied gratuitously with proper remedies. Thousands applied, who would otherwise have waited to *get ill* before going to a hospital to be treated. Who can deny that it would be well to *frighten* the people, if need be, into this degree of precaution? Without it you may rest assured there is no safety.

I deem it unnecessary to enter into a minute detail of the symptoms that characterize cholera, as they are familiar to most persons, whether belonging to the medical profession or not. It may not be amiss, however, to mention a few of the most remarkable ones that attended the late epidemic. Many bad cases were marked by an obstinate vomiting of bile, which continued until death. The vomiting would continue for days, and incredible quantities would be thrown up. In these cases the diarrhœa was generally moderate and sometimes absent. The worst cases were those in which the rice-water discharges were profuse, as well from the stomach as the bowels. In these, there was no appearance of bile whatever.

A few cases were seen at the Charity Hospital which terminated in black vomit.

When collapse supervened early in the attack, before the system was too much exhausted by copious rice-water evacuations, it was less difficult to bring about reaction, and there was better hope of ultimate success. So far as I know, the only cases of recovery that took place after decided collapse were of this kind.

Where reaction from a state of collapse was slow and difficult, a sort of typhus fever supervened, which lasted for some days, and generally terminated fatally with affection of the brain. The intellect was generally clear and undisturbed to the last, excepting in those who died of the consecutive fever. It is marvellous and astounding to witness the mental clearness and composure of some persons dying with cholera! Whilst the attendant relatives and friends are agonized with grief at the sudden and awful calamity, the poor victim is often seen supernaturally calm and uttering words of consolation with the expiring breath.

The treatment of cholera admits of much variation. Educated physicians everywhere concur in the indications to be fulfilled, or what is wanted to be done; but amidst the multiplicity of remedies at their command, of course each one resorts to such as he thinks are best adapted to the circumstances of the case. Doubtless the same object may be accomplished by a variety of means, if it can be accomplished at all; and in our choice of remedies we must be guided by observation and experience. In the treatment of what are called the premonitory symptoms, the first indication is—to check the diarrhœa as soon as possible, keeping an eye, at the same time, to the important secretions of the liver, kidneys and skin. For this purpose, physicians very generally resort to opium and its preparations, combined with stimulants and aromatics—or opium with some mercurial, or with quinine. According to the urgency of the symptoms a good prescription may be made of laudanum or paragorick and brandy—or laudanum, spirits of camphor and tincture of assafœtida—or laudanum, essence of peppermint and compound spirits of lavender—or calomel, opium and capsicum or camphor—or equal parts of paregorick and tincture of catechu. The following is a recipe which I found to answer very well, viz: ℞. Sulph. Quinin. ʒi, Tinct. Opii, ʒiss, Tinct. Capsici Comp., ʒiii, Mucilag. Acacia with Aqua Cinnamon, f. ℥iv., M. Give a table spoonfull and repeat after every two loose stools. Although opium is so often found in the prescriptions above, I should mention that some physicians disapprove of it and seldom prescribe it in cholera, except by enema. These gentlemen place their chief reliance upon calomel, combined with camphor, capsicum and the like. As to the sulphate of quinine, it appears to be serviceable in almost every kind of disease that occurs in this region. It possesses remarkable intrinsic virtues, and may be used as an adjuvant to many other remedies.

In the treatment of the violent or acute stage of cholera, when there is vomiting, purging and cramps, we resort to anodynes, antispasmodics, stimulants, calomel, &c., internally, aided by sinapisms, stimulating frictions, &c., externally.

The treatment of the stage of *collapse* is altogether desperate. As before stated, a vast majority of patients die after getting into this condition; and it is no wonder, for the system is then completely drained of its vital fluid. An excessive hemorrhage from a divided artery would not produce a greater prostration than is brought about by the copious serous evacuations of cholera; for all the fluid discharged is abstracted from the blood. Various remedies are used in this stage, such as sinapisms, blisters, the hot and the cold bath, the hot air bath, calomel, carbonate of ammonia, &c, &c.

The most remarkable recovery from collapse that I witnessed was effected by very large doses of calomel, washed down with tablespoon doses of laudanum, aided by sinapisms and frictions with spirits of turpentine and sweet oil. This was a lady who was rescued from the very jaws of death. She was afterwards pretty badly salivated, but recovered without serious injury.

Two other physicians attended her with me. In the case of Dr. Morgan, mentioned before, I witnessed the astonishing power of cold water in bringing about reaction from a hopeless state of collapse. Warmth was restored, the pulse returned at the wrist, and life was prolonged two or three days; but still it failed, for the injury sustained was irreparable. The cold bath was administered in this instance at the suggestion of my friends, Dr. Richardson, of Vicksburg, and Dr. Gustine, late of Natchez, who said they had derived great benefit from it in the cholera of 1832. Being favorably impressed with the power which the remedy displayed in the case of Dr. M., I resorted to it in two other cases of collapse in private practice. It produced reaction, but they both died for want of vital power to sustain it.

I must apologise for the length to which this communication has unexpectedly been drawn. I have not gone into the minutiæ of the theory and practice, or the pathology of cholera. It is to be hoped that our medical journal will be enriched by some valuable papers on these subjects, especially the pathology of the disease, which has been laboriously investigated by some of our most respectable physicians.

Before closing, I will offer a remark or two on the course to be pursued by persons exposed to the epidemic influence of cholera:

1. Let them avoid imprudent excesses of all kinds.

2. Let them not make too sudden and great a change in their established habits.

3. Most persons should avoid fish and oysters; also, acid fruits and vegetables, with the exception of rice, potatoes, and beans.

4. When feeling weak and slightly indisposed, let them take a little good brandy or wine.

5. Let them pay prompt attention to the first and slightest premonitory symptoms. Their family physician, or some respectable regular physician will give them the best advice they can obtain, as it is their interest as well as their duty to preserve the lives of their employers.

6. When some simple remedy does not quickly arrest the disease, they should send for their physician; they will be apt to do this sooner or later, and it is but right that he should have a fair chance to save them, as his reputation is involved in the result.

By attending to these simple directions, many may escape the impending danger; whilst, by neglecting them, thousands will fall into untimely graves.

Very respectfully,

New Orleans, Jan., 1849. E. D. FENNER.

